# Surgical Technique: “Perforate and Fill” Technique of Bone Grafting for Scaphoid Fracture Fixation

**DOI:** 10.1016/j.jhsg.2024.12.007

**Published:** 2025-01-30

**Authors:** Mehek Gupta, Heng Qi Hui Bernice, Mala Satkunanantham, Tina Munn Yi Lee, Xu Jieying, Lam-Chuan Teoh

**Affiliations:** ∗ Department of Hand and Reconstructive Microsurgery, Tan Tock Seng Hospital, Singapore, Singapore

**Keywords:** Bone graft, Nonunion, Scaphoid fracture

## Abstract

**Purpose:**

We introduce the “Perforate and Fill” technique for bone grafting of scaphoid fracture with delayed union and nonunion, which preserves the cartilage shell and does not break open the fibrous nonunion. This article describes the technique and reports the experience in 11 scaphoid fractures.

**Methods:**

The records of 11 patients whose scaphoid fractures were managed surgically with this bone grating technique from our institution from July 2017 to June 2024 were reviewed retrospectively. Patient and fracture factors, radiographic measurements of bone defect, postoperative films, and subjective and objective outcomes were considered.

**Results:**

The radiographic union of the fracture ranges from 36 to 110 days (an average of 68 days). In the last follow-up review, the affected wrists were pain free in all cases, and they were able to resume their premorbid status of vocation and resumed all physical activities. In nine cases, their total wrist motion (including flexion, extension, radial, and ulnar deviation) measured 130° to 195° (an average of 172° and 93% of the opposite wrists). In these nine cases, their grip strengths measured 28–50 kg (an average of 31.5 kg and is 97% of the opposite hand).

**Conclusions:**

In conclusion, in treatment of scaphoid fracture delayed union and nonunion, the “Perforate and Fill” technique of bone grafting is a good alternative to the conventional wedge grafting technique. The advantage of keeping the intact cohesive union of cartilage shell and a less-invasive approach may contribute to the success of the fracture union in our 11 cases using this technique.

**Type of study/level of evidence:**

Therapeutic IV.

The scaphoid bone is the most commonly fractured carpal bone, and up to 40% are undiagnosed at the initial presentation.^1^^,^^2^ A total of 5% to 10% of all scaphoid fracture cases progress to nonunion.^3^ Conventional terms define nonunion at 6 months postinjury, and delayed union as the time up to 6 months.^4^ Delayed union occurs because of bone gaps formed as a result of increased bone turnover at fracture sites, which causes bone resorption and regional bone loss. Displacement of articular fragments in a scaphoid fracture may also allow synovial fluid to pass between them and delay healing.^5^

Delayed union and nonunion of the scaphoid fractures with bone loss are indicated for bone grafting and internal fixation. The standard approach is the open surgical approach. A bone defect of >2 mm in width is considered an indication for bone graft, as the intrinsic compression of the screw may not be able to effectively close the gap, and bone grafting is hence indicated.^6^ Bone graft donor options include iliac crest, distal radius, and olecranon.^6^ A popular technique of bone grafting used is the wedge grafting technique described by Fisk and Fernandez. The wedge grafting involves opening into the fracture line to access the bone defect cavity, refashioning the fracture site with removal of the near side intervening cartilage shell to fit in the wedge graft.^7^ However, the arthroscopic approach is also gaining in popularity.^8^^,^^9^ The aim of the surgery for scaphoid fracture is for the eventual union of the fracture. Ferguson’s systematic review of 5,464 scaphoid fracture outcomes suggests a mean union rate of 80% for nonvascularized bone grafts.^10^

We have noticed from our experience that the cartilage shell in many of our cases of delayed and nonunion remained in continuity. This finding has been described as scaphoid fibrous nonunion.^11^^,^^12^ The bone defect cavitation looks substantially large on imaging, but in most of these cases, intraoperatively, there was good cohesion of the two scaphoid fragments, and the scaphoid moved as a single piece.

We developed the open scaphoid fracture fixation with “Perforate and Fill” technique of bone grafting. The technique preserves the cartilage shell in a displaced fracture and avoids breaking open the inherent cohesion union of the cartilage shell in a fracture with fibrous nonunion. We are describing the technique and reporting the experience in 11 scaphoid fractures.

## Materials and Methods

Records of patients, whose scaphoid fractures were managed surgically with this bone grating technique from our institution from July 2017 to June 2024 were reviewed retrospectively. A total of 111 patients in our institution underwent surgical fixation of scaphoid fractures during this time frame, of which 11 patients had nonunion or delayed union with intact cartilage shells and subsequently were included in the study. This technique can be attempted for fractures where the cartilage shell is adequate, even if the fracture is widely displaced, although might be challenging. If, upon initiation of surgery, there is no intact cartilage shell, the fracture may be fixed using other surgical techniques.

Headless compression screws from AutoFIX Stryker, Medartis-Aptus, and Depuy Synthes Johnson & Johnson were used in all our cases. Screw diameters ranging from 2 to 2.5 mm were used in these fixations.

Radiographic measurements were taken to measure the size of the bone defect cavitation on the delayed and nonunion scaphoid fractures. The length (L) and height (H) of these ellipsoidal cavitations were measured in millimeters (mm) and calculated into the area in square millimeters (sq mm) of the defect using the ellipsoidal formula of A = πLH. These parameters were obtained from our *hospital radiology online integrated image and information system*, which allows relevant lines to be drawn to obtain the width and length measurement. These measurements were assessed by two independent reviewers (T.L.C. and M.G.) to improve objectivity. In cases of ambiguity, the third reviewer was consulted. Fracture union was assessed radiologically as a stable implant and evidence of bridging trabeculae across the fracture line and clinically as a nontender fracture site.

Institutional review board approval was obtained for the study.

### Surgical technique

The patient is positioned supine with the affected arm on a hand table. The surgery is performed under general anesthesia or regional nerve block with arm tourniquet exsanguination.

With a predetermined decision for bone grafting based on radiological features, a cancellous bone graft is harvested from the ipsilateral olecranon via a technique described by the senior author.^5^ Approximately 3–5 mL of cancellous bone graft may be harvested and kept in a moist gauze for later use.

**Figure 1:**
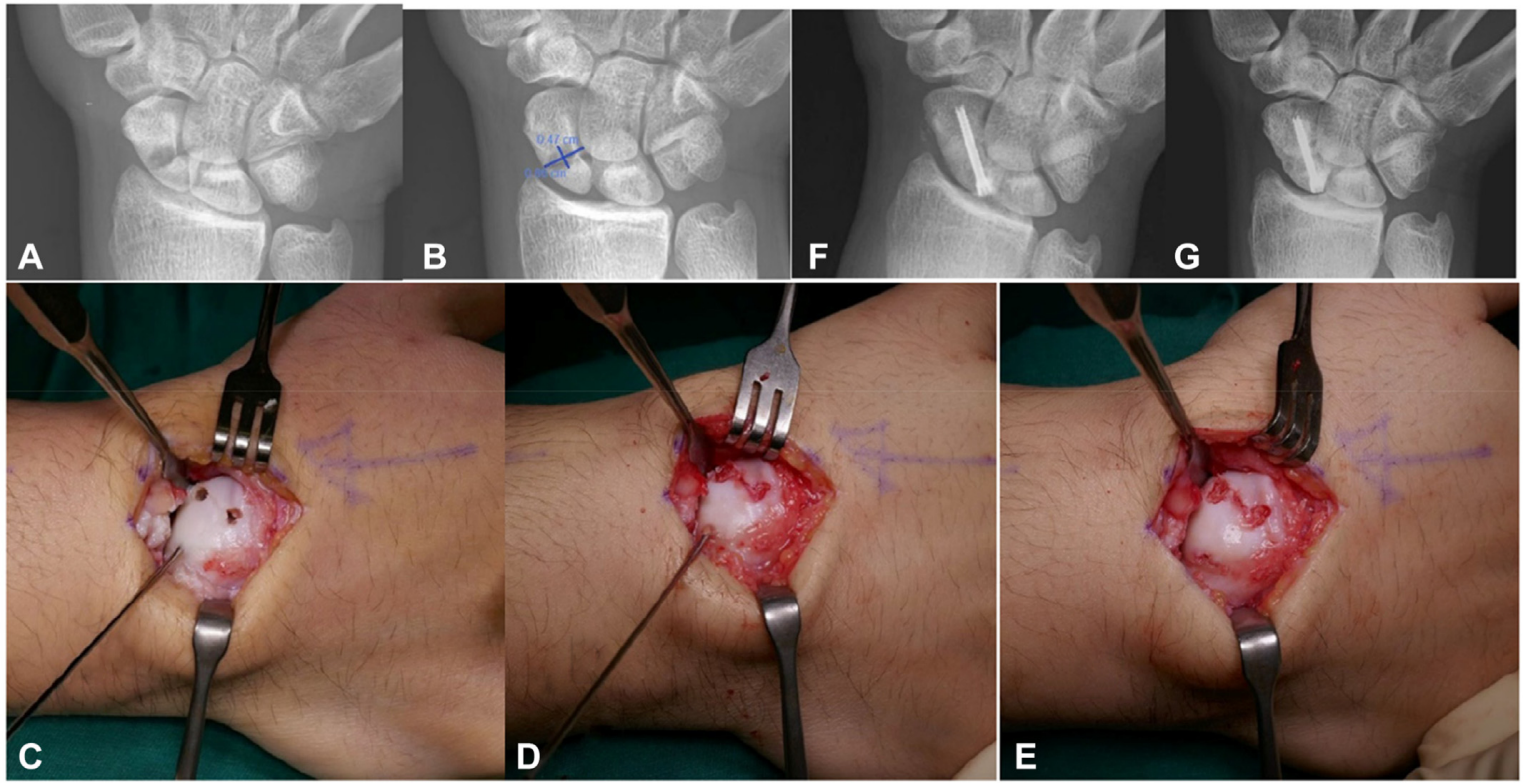
Case no 7. Scaphoid fracture bone grafting “Perforate and Fill” technique. (**A**) Radiograph showing proximal pole scaphoid fracture with radiolucent ellipsoidal cavitation from bone resorption. (**B**) Showing the size of cavitation measuring L = 9.4 mm and H = 4.7 mm, with an area of 139 sq mm and estimated volume of 1.7 mL. (**C**) Showing good cartilage cohesion of a fibrous nonunion, placement of the headless compression screw guide wire, and access to the fracture cavity with two 4 mm burr holes. (**D**) Morselized bone grafts were filled into one of the perforations in small batches, and further compacted in with a small bone tamp. (**E**) A predetermined headless compression screw of the chosen size and length is inserted through the guide wire till well seated deep to the cartilage. (**F**) Postoperative radiograph at 76 days showing a well filled cavitation and union of the fracture. (**G**) Radiograph 17 months postfixation showing a well united fracture of the scaphoid.

The fracture is exposed through either a palmar or dorsal approach based on the location of the scaphoid fracture site. The fracture site is identified and assessed for displacement, stability, integrity of cartilage shell, and cohesiveness of the two fragments. In cases of fibrous nonunion with intact cartilage shell and good cohesiveness of the two fragments, no attempt is taken to break open the fragments to access the fracture cavity. Fixation of the fracture commences with insertion of the cannulated scaphoid screw guide wire (one wire or two guide wires as predetermined) under fluoroscopy control. Two perforating holes over the fracture line are created using a small 4 mm burr to reach the fracture cavity, and the holes are placed as far apart as exposure allows. The cavity preparation and bone grafting are performed meticulously. A small 3 mm burr is walked radially over the wall of the cavity to gently push the content of the cavity out of the second burred hole. The bone grafts that were harvested were morselized and fed in small batches into one of the burr holes, pushed radially into the cavity with the blunt end of a 2.5 mm (0.098 in) K-wire, and further impacted with small bone tamp. The bone grafts should fill the second burr hole as well. If they are not, further filling of the bone graft through the second burr hole is performed (Fig. 1).

The final fixation of the fracture is performed with a predetermined length of one or two screws inserted through the previously prepared guide wire. The wound is then closed in layers, and the wrist is partially immobilized on a bulky dressing for 1 week. The wrist is further partially immobilized on a wrist brace, allowing interval non–weight-bearing active range of motion for a total of 6 weeks. Non-weighting activities are enforced till radiological union is achieved.

**Figure 2:**
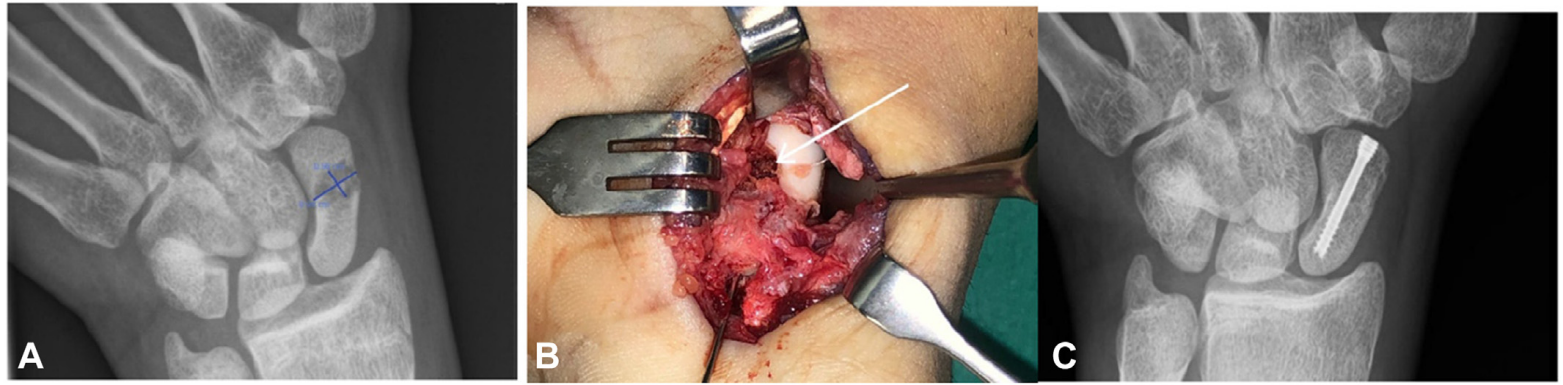
Case no 1. Distal pole fracture with palmar approach for “Perforate and Fill” bone grafting technique. (**A**) Radiograph showing the distal pole fracture with a large cavitation of L 9.4 mm × H 5.6 mm. (**B**) Surgical picture showing the fracture line is over the cartilage and fibrous part of the scaphoid, placement of the guide wire, and the white arrow showing the 4 mm burr hole access to the fracture cavity and bone grafts filling the cavity. (**C**) Radiograph showing sound union of the fracture.

In the palmar approach for the distal and waist fracture, the fracture line may be over the noncartilage-covered part of the scaphoid. For these cases, the access holes are similarly made over the fracture line. The cavity preparation, bone grafting, and fixation proceed in a similar manner without separating the fracture fragments (Fig. 2).

**Figure 3:**
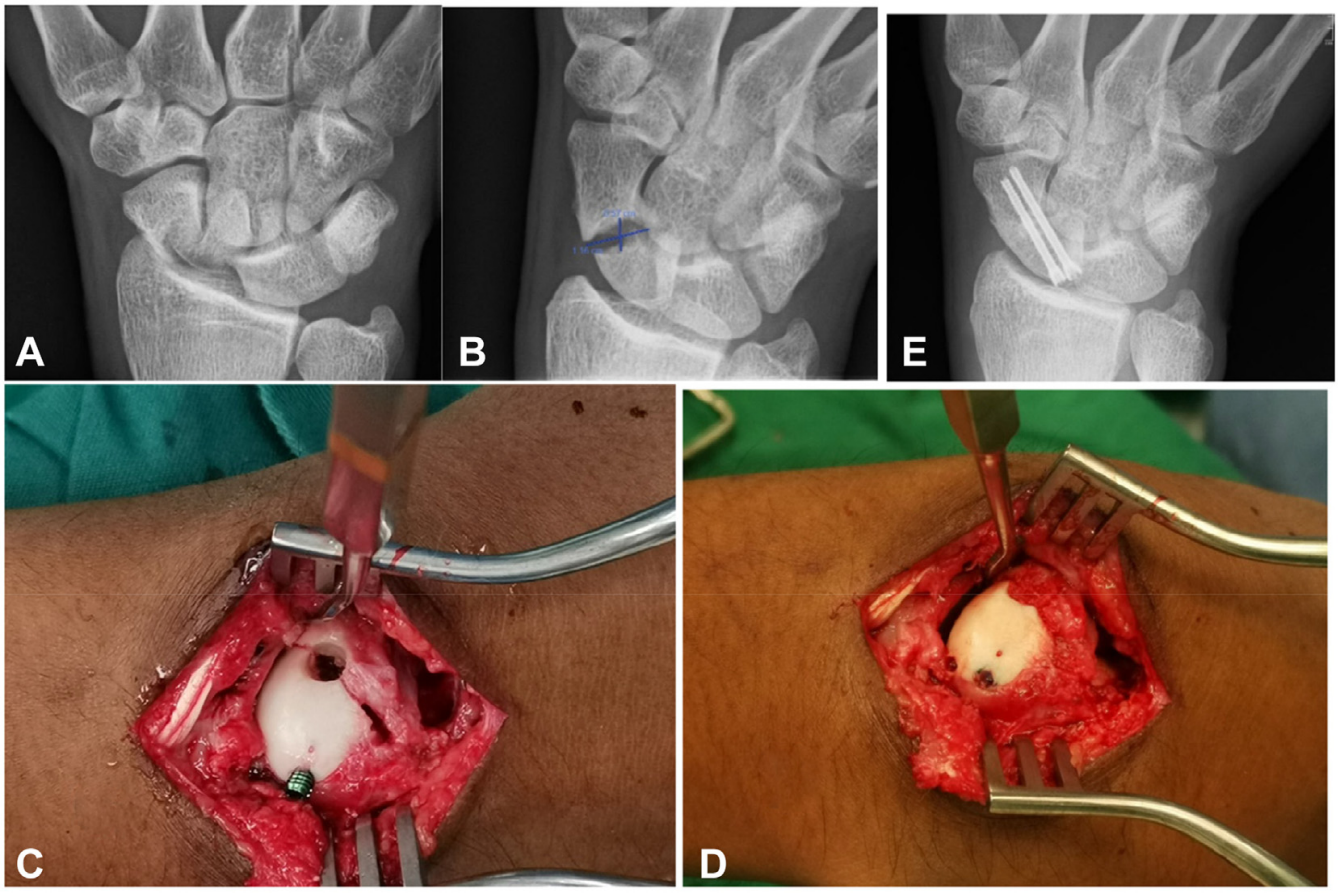
Case no 4. Waist fracture with unstable and widely displace fragment for “Perforate and Fill” bone grafting technique. (**A**) Radiograph showing widely displaced unstable fracture nonunion. (**B**) Radiograph showing fracture cavitation of L 11.6 mm × H 5.7 mm. (**C**) intraoperative picture showing reduction of the fracture, interim stabilization of the fracture with partial fixation, and the 4 mm burr holes to access the fracture cavity. (**D**) Showing the cavitation being filled with bone grafts and compacted and showing the final completion of the fixation.

In cases of displaced or separated fragments, the opportunity of access to the fracture is used to clean the cavity while preserving the cartilage shell. The fracture is reduced and partially fixed to close the fracture gaps. The access to bone grafting is similarly performed with two 4 mm burr holes. After completion of the bone grafting, the fixation is completed to further compress the fracture (Fig. 3).

**Figure 4:**
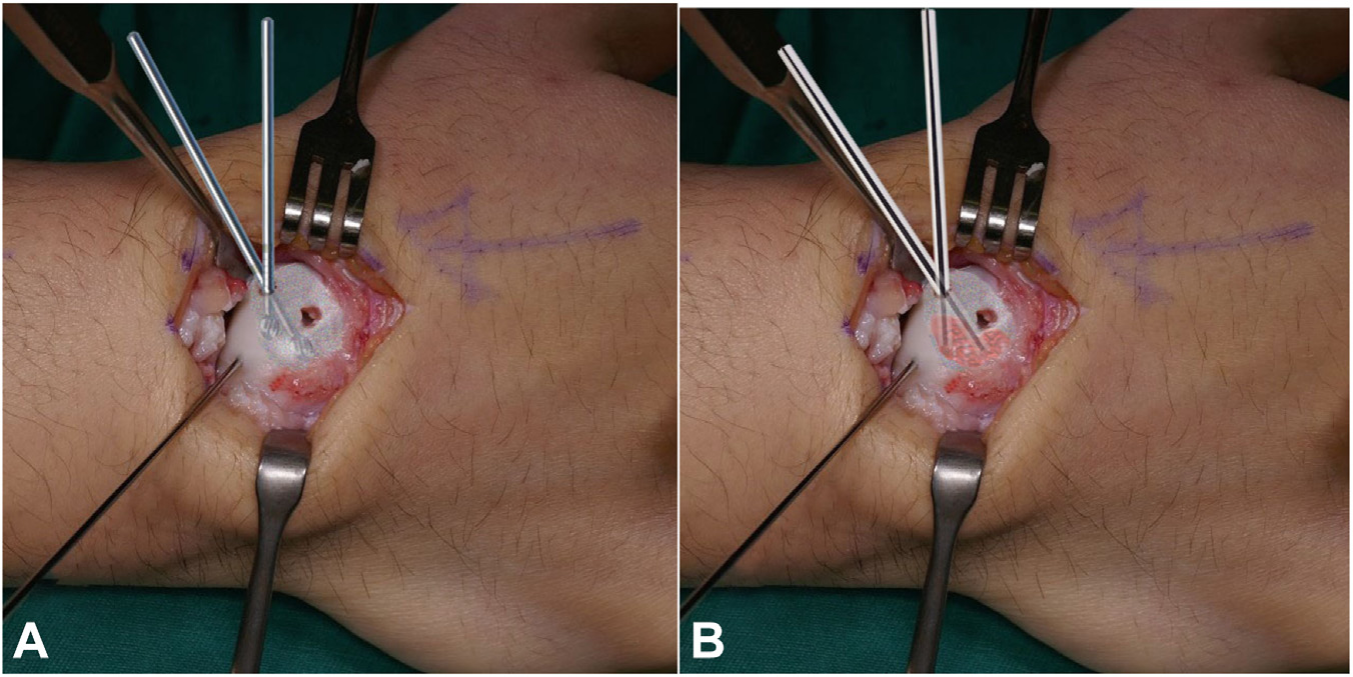
Illustration for case no 7 showing the burring and bone graft filing of the nonunion cavitation. The nonunion cavitation is represented by the layered area. (**A**) Showing the cavitation is gentle walked over with a 3 mm burr. (**B**) Showing the cavitation is filled with morselized bone graft from deep to superficial using the blunt end of a 2.5 mm K-wire.

Figure 4 provides a visual representation of how the cavitation can be burred and filled.

## Results

Our patients were relatively young, with ages ranging from 16 to 44 years old (an average of 24.7 years old). Nine cases were male and two were female. Eight of the fractures were in the right wrists, and three in the left wrists. Seven patients sustained fractures from sports, two from motor vehicle accidents, and another two from falling on outstretched hand. From the day of injury to the diagnosis of the delayed union or nonunion ranges from 32 to 219 days (an average of 89 days). Nine patients had no immobilization treatment, and two had scaphoid cast treatment for 2.5 and 4 months. Five fractures were at the waist, four at proximal pole, and two at the distal pole. Nine of the fractures were of the transverse type, and two were horizontal oblique type (Table 1).

**Table 1:** Preoperative Evaluations∗

Case No.	Sex/Age (y)/Occupation	Side of Wrist/Injury Mechanism	Interim Treatment	Days From Injury to Diagnosis	Fracture Location/Fracture Type	Size of Cavitation Length × Height in Millimeter (mm)	Area of Cavitation/Estimated Volume
1	M/20/intern	L/sports	None	32	Distal pole/transverse	9.4 × 5.6	165 sq mm/2.1 mL
2	M/ 30/tool maker	R/MVA	None	53	Waist/horizontal oblique	7.0 × 5.5	121 sq mm/1.1 mL
3	M/21/police	R/sports	None	100	Waist/transverse	8.6 × 5.8	157 sq mm/1.8 mL
4	M/20/national service	R/sports	None	48	Prox pole/horizontal oblique	11.6 × 5.7	261 sq mm/3.2 mL
5	M/18/student	R/sports	Cast Rx 4 mo	120	Prox Pole/transverse	8.8 × 3.2	88 sq mm/1.0 mL
6	F/44/barista	R/FOOSH	None	219	Waist/transverse	8.9 × 3.8	106 sq mm/1.3 mL
7	M/17/student	R/sports	None	34	Prox Pole/transverse	9.4 × 4.7	139 sq mm/1.7 mL
8	M/21/lawyer	R/sports	None	156	Prox Pole/transverse	8.4 × 4.7	124 sq mm/1.4 mL
9	M/37/construction	L/FOOSH	None	52	Distal pole/transverse	9.5 × 5.5	164 sq mm/2.1 mL
10	M/21/police	R/sports	None	84	Waist/transverse	8.8 × 5.5	152 sq mm/1.8 mL
11	F/27/entrepreneur	L/MVA	Cast Rx 2.5 mo	84	Waist/transverse	9.3 × 2.5	73 sq mm/0.9 mL

FOOSH, fall on outstretched hand; MVA, motor vehicle accident.

∗ Injury mechanism by MVA and FOOSH.

The size of the bone resorption ellipsoidal cavitation ranges from 7 to 11.6 mm in length (L) and 2–5.8 mm in height (H). These measurements were calculated for ellipsoidal area using the formula A = πLH, and the area of the cavitation ranges from 73 sq mm to 261 sq mm. Assuming the scaphoid is close to tubular in shape, the volume of the defect is calculated with a formula of V = 4/3 πLLH. The estimated volume of these cavitation ranges from 0.9 to 3.2 mL (an average of 1.67 mL; Table 1).

Dorsal approach to the scaphoid was used in seven cases, and palmar approach in four cases. All 11 scaphoids had well preserved healthy normal-looking cartilage. In 10 cases with fibrous nonunion, the cartilage shell had a firm cohesion at the fracture site, the scaphoid moves in one piece and is inseparable with some force applied to the fracture site. Only in case 4, a fracture of horizontal oblique type was unstable and widely displaced. Seven scaphoids were fixed with a single screw, and four scaphoids were fixed with two screws. The radiographic union of the fracture ranges from 36 to 110 days (a mean of 68 days). As many of our patients reside in other countries, the follow-up period ranges from 4 to 17 months (an average of 6 months). In the last follow-up review, the affected wrists were pain free in all cases, and they were able to resume their premorbid status of vocation and resume all physical activities. In nine cases, their total wrist motion (including flexion, extension, radial, and ulnar deviation) measured 130° to 195° (an average of 172° and that outlives the slow bone healing 93% of the opposite wrists). In these nine cases, their grip strengths measured 28–50 kg (an average of 31.5 kg and is 97% of the opposite hands; Table 2).

**Table 2:** Surgical findings and results

Case No.	Surgical Approach	Intraoperative Findings	Implants	Time to Union	Follow-Up Duration	Result: Pain Status, (RPVA), (TWM), Grip Strength (Kg)
1	Palmar	Stable fibrous nonunion	AutoFIX One 2.5 mm screw	36 d	16 mo	Pain free, (RPVA) yes, (TWM) L 175/R 180, Grip L 30/R 34
2	Palmar	Stable fibrous nonunion	Medartis-Aptus CCS two 2.2 mm screws	54 d	6 mo	Pain free, (RPVA) yes, (TWM) R 175/L 190, Grip R 50/L 50
3	Dorsal	Stable fibrous nonunion	HCCS one 2.5 mm screw	110 d	6 mo	Pain free, (RPVA) yes, (TWM) R 170/L 190, Grip R 30/L 34
4	Dorsal	Unstable and widely separated fracture	AutoFIX two 2.0 mm screws	80 d	7 mo	Pain free, (RPVA) yes, (TWM), Grip
5	Dorsal	Stable fibrous nonunion	Depuy Synthes HCS one 2.5 mm screw	43 d	5 mo	Pain free, (RPVA) yes, (TWM) R 195/L 210, G rip R 24/L 24
6	Palmar	Stable fibrous nonunion	Depuy Synthes HCS two 2.4 mm screw	44 d	6 mo	Pain free, (RPVA) yes, (TWM), Grip
7	Dorsal	Stable fibrous nonunion	Depuy Synthes HCS one 2.4 mm screw	76 d	18 mo	Pain free, (RPVA) yes, (TWM) R 180/L 180, Grip R30/L 28
8	Dorsal	Stable fibrous nonunion	Depuy Synthes HCS one 2.4 mm screw	90 d	17 mo	Pain free, (RPVA) yes, (TWM) R 190/L 190, Grip R 32/L 28
9	Palmar	Stable fibrous nonunion	Medartis-Aptus CCS two 2.2 mm screws	54 d	6 mo	Pain free, (RPVA) yes, (TWM) R 165/L 180, Grip R 30/L 32
10	Dorsal	Stable fibrous nonunion	Depuy Synthes HCS one 2.5 mm screw	110 d	6 mo	Pain free, (RPVA) yes, (TWM) R 170/L 190, Grip R 30/L 34
11	Dorsal	Stable fibrous nonunion	Depuy Synthes HCS one 2.5 mm screw	54 d	4 mo	Pain free, (RPVA) yes, (TWM) L 130/R 160, Grip L 28/R 30

CSS, cannulated screw system; HCS, headless compression screw; RPVA, resuming all premorbid vocation and activities; TWM, total wrist motion in degrees (flexion+ extension+ radial deviation+ ulnar deviation).

## Discussion

The goal of treatment for a fractured scaphoid is to achieve a bony union within a reasonable time frame. A fractured scaphoid in the stage of delayed union or nonunion will have bone resorption that frequently presents on X-rays as an ellipsoidal cavitation. These ellipsoidal cavitations are surprisingly large in our 11 cases estimated to be from 0.9 to 3.2 mL (an average of 1.67 mL). The scaphoid does not always have a gap at the site of the fracture, and there may be good cohesion of the two fracture fragments with substantial stability at the fracture site.^10^^,^^11^ The cartilage of the scaphoid is typically in good condition as it survives on synovial nutrition.

The treatment for delayed union and nonunion of a scaphoid fracture is bone grafting of the bone defect and stable fixation. The healing of the fracture is by the slow process of creeping substitution from both ends of the scaphoid through the bone graft. Therefore, good compacted bone grafting and stable fixation that outlives slow bone healing are necessary. The method of fixation of the scaphoid with headless compression screws is well-developed and universally accepted. However, the approach to bone grafting varies widely. The Fisk Fernandez technique involves undoing the cohesive union at the fracture site, removal of some good cartilage shell, and reconstituting the ensuing bony defect with a bone graft devoid of cartilage protection^6^ and technically requires good refined skills to do the procedure well. A less-invasive approach is to open into the fracture site and do an inlay bone grafting, sacrificing the cohesive stability and a less compact bone grafting. The arthroscopic assisted bone grafting approach is to access the fracture cavity with a trough burred over a length of the fracture and keeping much of the fracture line undisturbed.^7^^,^^8^

The “Perforate and Fill” technique preserves the cartilage shell at the fracture site. The access to the fracture cavity is through two 4 mm burr holes. We believe the intact cartilage shell helps achieve a denser and more compacted bone grafting. To our surprise, the cohesive stability of the fibrous nonunion at the fracture site was relatively strong, and the process of cleaning the fracture cavity and compaction of the bone graft did not separate the fragments. In our experience, the intact cartilage shell contributes to stability of the fixation and certainly helps contain the bone graft. Keeping the cartilage shell intact and the access for grafting to two small perforations help keep out the synovial fluid that may interfere with this slow process of bone healing.

However, the study does have limitations. It does not use more detailed radiological tools such as computed tomography or magnetic resonance imaging to delineate the fracture pattern prior to and after fixation, as not all patients had such imaging performed. Furthermore, the patients discussed have variable follow-up timelines, and this limits the identification of an average time to union. This study is retrospective, and a detailed prospective study with a larger number of patients, variable patient demographics, and fixed follow-up timeline would be optimal in making generalized conclusions about the efficacy of the technique. This article also does not address scaphoid malunion such as humpback deformity, as it only addresses fractures that had fibrous nonunion with bone gaps.

The “Perforate and Fill” technique is technically much simpler and reproducible, as the cartilage shell remains intact, and it is easier to handle the scaphoid in a single piece. The access to the fracture cavity may be limited, restricting the cleaning of the cavity more thoroughly and distributing the bone graft more evenly. Our 11 cases (including four cases of proximal pole fractures) all went into union before 6 months, the longest patient taking 110 days for radiographic union. We believe the limited access to fracture sites with this technique may not be an issue.

## Conflicts of Interest

No benefits in any form have been received or will be received related directly to this article.
